# Clinical predictors of moderate-to-severe pediatric obstructive sleep apnea

**DOI:** 10.3389/fped.2024.1421467

**Published:** 2024-10-25

**Authors:** Kantarakorn Unchiti, Artid Samerchua, Tanyong Pipanmekaporn, Prangmalee Leurcharusmee, Nuntigar Sonsuwan, Phichayut Phinyo, Jayanton Patumanond

**Affiliations:** ^1^ Department of Anesthesiology, Faculty of Medicine, Chiang Mai University, Chiang Mai, Thailand; ^2^ Department of Otolaryngology, Faculty of Medicine, Chiang Mai University, Chiang Mai, Thailand; ^3^ Center for Clinical Epidemiology and Clinical Statistics, Faculty of Medicine, Chiang Mai University, Chiang Mai, Thailand; ^4^ Department of Family Medicine, Faculty of Medicine, Chiang Mai University, Chiang Mai, Thailand; ^5^ Musculoskeletal Science and Translational Research (MSTR), Faculty of Medicine, Chiang Mai University, Chiang Mai, Thailand

**Keywords:** pediatric, obstructive sleep apnea, obstructive sleep disordered breathing, risk factor, predictor

## Abstract

**Background:**

Children with moderate-to-severe obstructive sleep apnea (OSA) require specific management. However, the risk factors associated with this level of severity in pediatric OSA remain poorly defined. This study aimed to identify clinical predictors of moderate-to-severe pediatric OSA.

**Methods:**

This retrospective study enrolled children aged 1–18 years who underwent respiratory polygraphy or nocturnal oximetry between January 2013 and December 2017. Patient history, demographics, and sleep study data were analyzed. Logistic regression analysis was conducted to assess risk factors associated with moderate-to-severe pediatric OSA. The STROBE checklist was followed in reporting this research.

**Results:**

Among 818 children with a median age of 5 years (IQR: 3, 9), 69.4% were male, and 96.7% were diagnosed with OSA. Of those diagnosed, 542 (66.3%) had moderate-to-severe cases. Independent predictors of moderate-to-severe OSA, with their adjusted odds ratios (95% CI), included: age 1–5 years; 6.16 (3.98–9.53), obesity; 2.08 (1.35–3.19), adenotonsillar hypertrophy; 1.58 (1.05–2.36), frequent snoring (>5 nights/week); 6.86 (4.40–10.67), stopped breathing during sleep; 2.34 (1.50–3.63), awakening during sleep; 2.04 (1.32–3.12), and excessive daytime somnolence; 2.10 (1.28–3.43).

**Conclusions:**

Children referred to a sleep center demonstrated an increased risk of being diagnosed with moderate-to-severe OSA. Key clinical predictors of moderate-to-severe OSA included age 1–5 years, frequent snoring, episodes of stopped breathing or awakening during sleep, excessive daytime somnolence, obesity, and adenotonsillar hypertrophy. Prioritizing early sleep investigations and appropriate management for children exhibiting these predictors may enhance clinical outcomes and mitigate the risk of long-term complications.

## 1 Introduction

Obstructive sleep disordered breathing (SDB) is a syndrome characterized by upper airway dysfunction during sleep, resulting in snoring and/or increased respiratory effort due to increased upper airway resistance and pharyngeal collapsibility. Obstructive sleep apnea (OSA) is a subset of SDB, characterized by recurrent episodes of partial or complete upper airway obstruction (hypopneas, obstructive or mixed apneas), leading to disruptions in normal oxygenation, ventilation, and sleep patterns (1).

OSA severity varies among individuals and has significant impacts on health, including neurocognitive function, cardiovascular health, growth, and overall quality of life (2). The prevalence of pediatric OSA ranges from 1% to 4% in the general populations (3). Many children with OSA undergo anesthesia and surgery, even when the procedure is unrelated to otolaryngology. Unfortunately, a significant number pediatric OSA cases go unrecognized preoperatively, exposing patients to an increased risk of perioperative complications (4).

To ensure optimal care for pediatric OSA, understanding the risk factors associated with disease severity and their impact on health outcomes, diagnostics, treatments, perioperative protocols, and long-term monitoring is crucial (5). Children with moderate-to-severe OSA experience higher incidences of complications and require intervention to resolve the condition, as spontaneous improvement is unlikely (1). Furthermore, individuals with moderate-to-severe OSA are at greater risk of respiratory complications during the perioperative periods, such as upper airway obstruction and desaturation, which may lead to unplanned admissions to the intensive care unit (6).

While various risk factors for pediatric OSA have been identified, such as adenotonsillar hypertrophy, allergic rhinitis, obesity, craniofacial abnormalities, and neuromuscular disorders, limited research has specifically focused on the risk factors associated with moderate-to-severe OSA in children (1). Given the significant impact of moderate-to-severe OSA on patient health outcomes, resource utilization, and clinical decision-making throughout the care continuum—from diagnostic workup to perioperative management and long-term follow-up—the identification of independent clinical predictors for this specific OSA severity subgroup is of paramount importance. Therefore, the objective of this study was to investigate the clinical predictors independently associated with moderate-to-severe OSA in children referred to a specialized sleep center and underwent sleep tests. By identifying these predictors, we aim to provide valuable insights for improving the identification and management of moderate-to-severe OSA in pediatric populations, ultimately optimizing care and resource allocation.

## 2 Materials and methods

### 2.1 Study design

This retrospective study was approved by the Research Ethics Board at Maharaj Nakorn Chiang Mai (ANE-2564-07976), Chiang Mai, Thailand. This research was reported in accordance with the STROBE guideline.

### 2.2 Setting and participants

Children aged 1–18 years suspected of having SDB due to upper airway obstruction, who had been evaluated by an otolaryngologist and underwent either respiratory polygraphy (RPG) or nocturnal oximetry between January 2013 and December 2017 were included in the study. Exclusions consisted of children with incomplete RPG or nocturnal oximetry findings and those who had previously undergone tonsillectomy and adenoidectomy.

### 2.3 Study variables

Children referred to the sleep clinic were asked about signs and symptoms associated with OSA, including regular snoring, episodes of apnea, awakening during sleep, and daytime somnolence (7). A physical examination related to OSA, assessing tonsil and adenoid size, was performed. Those children who snored regularly or exhibited signs and symptoms of OSA were subsequently scheduled for sleep studies.

Patient medical history data was obtained from our center's electronic medical record system. Frequent snoring was defined as occurring on more than five nights per week (8). Excessive daytime somnolence (EDS) was defined as frequently experiencing excessive sleepiness during the daytime (9). Patient classification for specific conditions adhered to established criteria. Positive status for allergic rhinitis was determined by a qualified healthcare provider's confirmed diagnosis. Documented use of anti-inflammatory medications for OSA treatment, such as intranasal steroids, oral montelukast, or antihistamines, indicated a positive anti-inflammatory treatment status. A formal attention-deficit/hyperactivity disorder (ADHD) diagnosis by a qualified physician established positive ADHD status.

The patient's physical characteristics, including height and weight, were collected. Obesity was defined as a body mass index (BMI) above the 95th percentile, adjusted for age and gender. Craniofacial abnormalities were identified as congenital deformities affecting the cranial and facial bones, such as orofacial cleft, Down syndrome, midface deficiency, and mandibular hypoplasia (10).

Tonsil dimensions were assessed by otolaryngologists using the standardized Brodsky scale (ranging from 0 to 4), with tonsillar hypertrophy defined as grade 3 or grade 4. Flexible nasopharyngoscopy was performed to assess adenoid size, four grades being used for categorization based on the percentage of obstruction: Grade I (0%–25%), Grade II (25%–50%), Grade III (50%–75%), and Grade IV (75%–100%) (11). Adenoid hypertrophy, defined as grades III to IV, can cause significant airway obstruction and related symptoms (12, 13). In this study, patients were classified as having adenotonsillar hypertrophy if they exhibited either grade 3 or 4 tonsillar hypertrophy in conjunction with grade 3 or 4 adenoid hypertrophy.

### 2.4 Clinical endpoints

Polysomnography (PSG) is considered the gold standard for diagnosing OSA (14, 15). However, in resource-limited settings such as Thailand, including our center, the high cost and limited availability of PSG often lead to extended waiting periods for testing. Therefore, RPG or nocturnal oximetry, conducted and interpreted by an experienced pediatric sleep specialist, is recommended for the diagnosis of pediatric OSA. RPG is a technique that monitors cardiorespiratory function such as airflow, chest and abdominal wall movements, pulse oximetry, the ECG and position without simultaneously assessing sleep and wakefulness (15). At our center, we conduct RPG using a portable sleep machine (SOMNOlab 2, Hamburg, Germany), to monitor parameters including the apnea-hypopnea index (AHI), oxygen desaturation index (ODI), oxygen saturation (SpO_2_) nadir and mean SpO_2_. OSA severity was classified as mild (AHI 1–4 events/hour), moderate (AHI 5–9 events/hour), and severe (AHI≥10 events/hour).

However, applying RPG in pediatric populations can be challenging due to difficulties in obtaining cooperation and potential discomfort in unfamiliar environments (16). For children weighing less than 20 kg or with limited cooperation, nocturnal oximetry was used as an alternative diagnostic method, providing data on maximum, average, and minimum levels of SpO_2_ (16, 17).

Regarding the diagnostic capability of nocturnal oximetry, the severity of OSA in this context was categorized using the McGill oximetry score (17). An inconclusive study was defined as having fewer than 3 desaturations below 90%, mild OSA was defined as having 3 or more desaturations below 90% but no more than 3 desaturations below 85%, moderate OSA was defined as having 3 or more desaturations below 85% but no more than 3 desaturations below 80%, and severe OSA was defined as having more than 3 desaturations below 80%.

### 2.5 Statistical methods

#### 2.5.1 Sample size estimation

The sample size for this study was determined using retrospective pilot data from 30 children (age 1–18 years old) suspected of having OSA in 2012. Various potential risk factors including obesity, snoring, breathing cessation, sleep disturbances, ADHD, EDS, tonsillar hypertrophy, adenoid hypertrophy, and craniofacial abnormalities were collected to estimate the required sample size. The sample size calculation was based on two independent proportion tests. Tonsillar hypertrophy required the largest sample size, with proportions of 0.3 in the no and mild OSA group and 0.43 in the moderate-to-severe OSA group, resulting in an N2 to N1 ratio of 2.0. With a significance level of 0.05 and a desired power of 80%, the calculated minimum sample size for the study was 522. The analysis ultimately included data from a total of 818 patients, providing a sufficiently large sample size for statistically significant results.

#### 2.5.2 Statistical analysis

Statistical analyses were conducted using STATA software version 16.0 (StataCorp LP, College Station, TX, USA). Categorical data were presented as frequencies and percentages and compared using the Chi-squared or Fisher's exact test. Numerical data were described as mean ± standard deviation (SD) or as medians and interquartile ranges (IQR) for variables with a skewed distribution. *T*-test or Wilcoxon ranksum test was employed for comparing normally or abnormally distributed data, respectively. Certain continuous variables were transformed into categorical data based on theoretical considerations. Age was categorized into two groups: 1–5 years and 6–18 years (18). Logistic regression analysis was performed to identify predictive factors for moderate-to-severe pediatric OSA, with odds ratios (OR) and their corresponding 95% confidence intervals (CI) reported. To address potential limitations and biases arising from the use of non-overlapping diagnostic methods between RPG and nocturnal oximetry, multivariable regression analysis was employed, enabling the control of confounding factors and the assessment of independent predictors of moderate-to-severe OSA. Variables with a univariable logistic regression *p*-value less than 0.2 or those theoretically associated with OSA were included in the multivariable logistic regression analysis. The final model included only those predictors with a *p*-value less than or equal to 0.05 in the univariable logistic regression analysis.

## 3 Results

**Figure 1:**
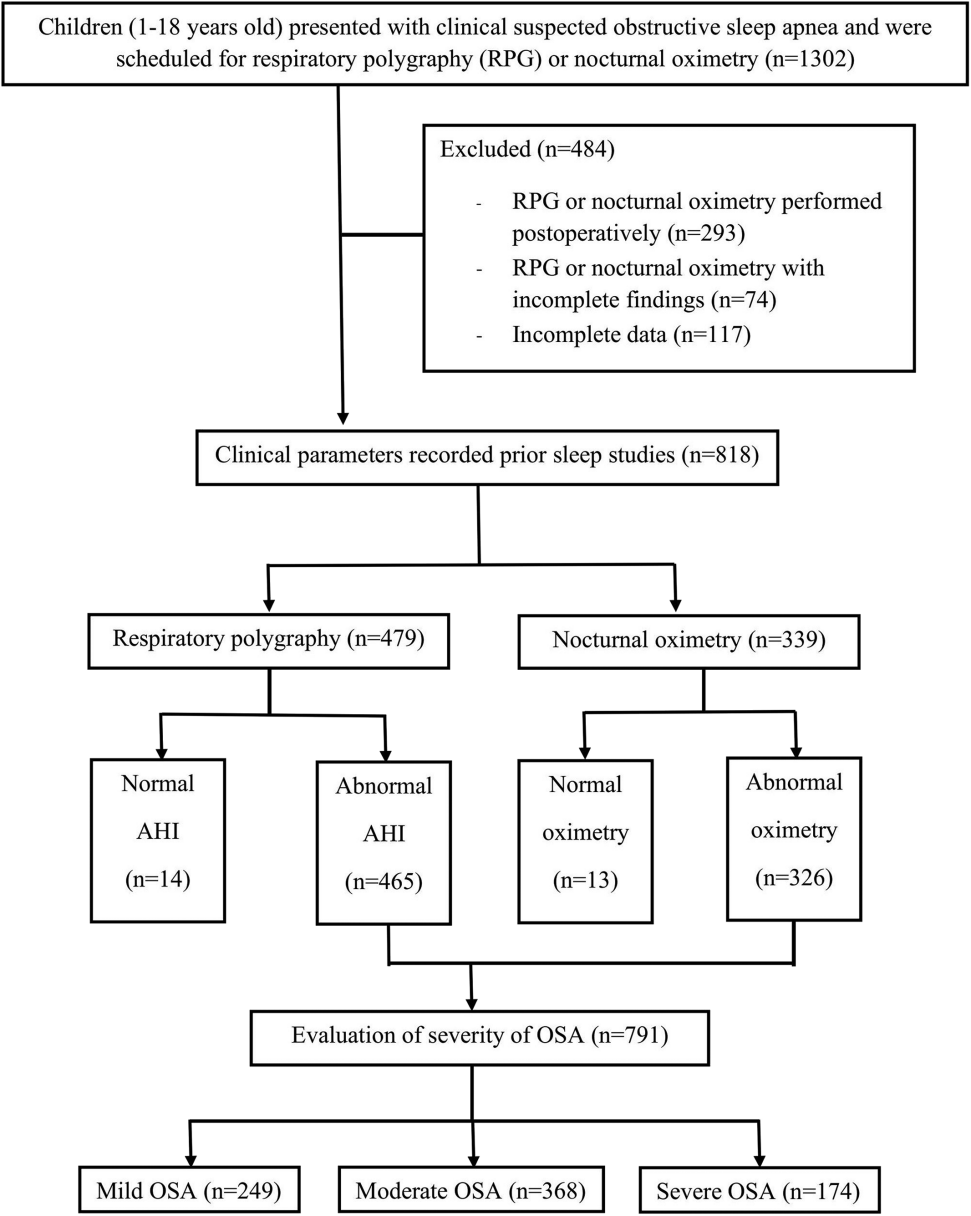
Study flow diagram.

The study initially enrolled 1,302 children; however, 484 were excluded due to undergoing postoperative sleep tests or having incomplete findings. Consequently, 818 patients were included in the final analysis, with 479 having undergone RPG and 339 nocturnal oximetry assessments (Figure 1). Among these, 791 (96.7%) were diagnosed with OSA. The cohort comprised 568 boys (69.44%) and 250 girls (30.56%), with a median age of 5 years IQR (3, 9). The prevalence of OSA was significantly higher in males compared to females (70% vs. 30%, *p* = 0.044). No significant differences were observed in the occurrence of OSA between the age groups of 1–5 years and older children (51% vs. 49%, *p* = 0.506).

Among the children with OSA, 249 (30.4%) had mild OSA, and 542 (66.3%) had moderate-to-severe OSA. The demographic and clinical data of children with no or mild OSA and those with moderate-to-severe OSA are illustrated in Table 1. Children with moderate-to-severe OSA were significantly younger than 5 years (*p* < 0.001) and had higher rates of obesity (*p* = 0.023) and adenotonsillar hypertrophy (*p* < 0.001) compared to those with no or mild OSA. Additionally, children with moderate-to-severe OSA exhibited more typical symptoms of OSA (*p* < 0.001). The prevalence of moderate-to-severe OSA did not significantly differ between males and females or in patients with craniofacial abnormalities. Furthermore, the use of anti-inflammatory drugs for the treatment of OSA was comparable between the two groups.

**Table 1:** Demographic and clinical characteristics of the 818 patients compared between those with moderate-to-severe OSA and those without or with mild OSA.

Characteristics	Moderate-to-severe OSA (*n* = 542)	No and mild OSA (*n* = 276)	*p*-Value
Age (years) [median (IQR)]	5 (3, 7)	8 (5, 10)	<0.001
Age 1–5 years (*n*,%)	345 (63.7)	70 (25.4)	<0.001
6–18 years (*n*,%)	197 (36.4)	206 (74.6)	
Sex Male (*n*,%)	381 (70.3)	187 (67.8)	0.456
Female (*n*,%)	161 (29.7)	89 (32.3)	
BMI^a^ (kg/m^2^) [median (IQR)]	17.1 (15, 22)	16.6 (15, 22)	0.481
Obesity (*n*,%)	217 (40)	88 (31.9)	0.023
Craniofacial abnormalities (*n*,%)	27 (5)	14 (5.1)	0.955
Adenotonsillar hypertrophy (*n*,%)	379 (69.9)	124 (44.9)	<0.001
Allergic rhinitis (*n*,%)	49 (9)	33 (12)	0.189
ADHD^b^ (*n*,%)	27 (5)	7 (2.5)	0.098
Anti-inflammatory treatment (*n*,%)	491 (90.6)	252 (91.3)	0.738
Common clinical symptoms of OSA			
Frequent snoring (*n*,%)	481 (88.7)	95 (34.4)	<0.001
Stopped breathing during sleep (*n*,%)	358 (66.1)	59 (21.4)	<0.001
Awakening during sleep (*n*,%)	307 (56.6)	57 (20.7)	<0.001
Excessive daytime somnolence (*n*,%)	174 (32.1)	47 (17.1)	<0.001

^a^ BMI, body mass index.

^b^ ADHD, attention deficit/hyperactivity disorder.

Table 2 presents the results of the univariable analysis, identifying age, obesity, adenotonsillar hypertrophy, and common clinical symptoms of OSA—such as frequent snoring, stopped breathing during sleep, awakening during sleep, and EDS—as potential predictors of moderate-to-severe OSA. To develop the final multivariable model, allergic rhinitis, ADHD, and craniofacial abnormalities were also included based on previous studies and investigator consensus (1, 2, 19–22).

**Table 2:** Univariable and multivariable logistic regression analysis for an independent predictor of moderate-to-severe OSA.

Characteristics	Univariable analysis				Multivariable analysis			
(*n* = 542)	uOR	95% CI		*p*-value	aOR	95% CI		*p*-value
Age 1–5 years	5.15	3.73	7.11	<0.001	6.16	3.98	9.53	<0.001
Allergic rhinitis	0.73	0.45	1.16	0.191	0.84	0.45	1.55	0.588
ADHD^a^	2.01	0.86	4.68	0.104	2.70	0.91	7.92	0.071
Obesity	1.42	1.05	1.93	0.023	2.08	1.35	3.19	0.001
Craniofacial abnormalities	0.98	0.50	1.90	0.955	1.29	0.52	3.18	0.578
Adenotonsillar hypertrophy	2.85	2.11	3.84	<0.001	1.58	1.05	2.36	0.025
Frequent snoring	15.02	10.4	21.6	<0.001	6.86	4.40	10.67	<0.001
Stopped breathing during sleep	7.15	5.10	10.0	<0.001	2.34	1.50	3.63	<0.001
Awakening during sleep	5.01	3.58	7.03	<0.001	2.04	1.32	3.12	0.001
Excessive daytime somnolence	2.30	1.60	3.30	<0.001	2.10	1.28	3.43	0.003

^a^ ADHD, attention deficit/hyperactivity disorder.

The multivariable analysis indicated several significant independent predictors of moderate-to-severe OSA: age 1–5 years (OR 6.16; 95% CI, 3.98–9.53), obesity (OR 2.08; 95% CI, 1.35–3.19), and adenotonsillar hypertrophy (OR 1.58; 95% CI, 1.05–2.36). Common clinical symptoms, including stopped breathing during sleep (OR 2.34; 95% CI, 1.50–3.63), awakening during sleep (OR 2.04; 95% CI, 1.32–3.12), frequent snoring (OR 6.86; 95% CI, 4.40–10.67), and EDS (OR 2.10; 95% CI, 1.28–3.43), were also significant predictors. This study revealed that children aged 1–5 years and those who snored more than 5 nights a week were at 6 times higher risks of moderate-to-severe OSA. Additionally, children who were obese, had adenotonsillar hypertrophy, reported stopped breathing or awakening during sleep, and experienced EDS had twice the risk of moderate-to-severe OSA than those with no or mild OSA.

## 4 Discussion

The majority of children referred for sleep testing exhibited symptoms of sleep-disordered breathing, which explains the high prevalence of OSA (96.7%) found in our study, with 66.3% of the children being diagnosed with moderate to severe OSA. These findings align with prior studies, which reported rates of moderate-to-severe OSA at approximately 80% in referral centers (22). Our study identified several risk factors for moderate-to-severe pediatric OSA, including age 1–5 years, obesity, adenotonsillar hypertrophy, and sleep-related symptoms such as frequent snoring, apnea, nocturnal awakenings, and EDS.

### 4.1 Age and gender risk factors

The influence of age on OSA prevalence and severity remains inconclusive. While our study found no significant effect of age on the overall prevalence of OSA, age was strongly correlated with OSA severity; notably, 83% of children aged 1–5 years had moderate-to-severe OSA. This finding is consistent with previous research conducted in referral centers, where preschool-aged children were more susceptible to moderate-to-severe OSA, most likely due to age-related variations in upper airway anatomy (22, 23). Children older than two years are particularly vulnerable due to factors such as adenotonsillar hypertrophy, neuromuscular disorders, and obesity, whereas infants (0–1 year) is often associated with craniofacial syndromes and prematurity (24).

Previous studies have identified male gender as a predictor of pediatric OSA, with boys showing a higher prevalence of moderate-to-severe OSA compared to girls, potentially due to a higher incidence of obesity, atopy, and allergic rhinitis (25, 26). However, in our study, gender was not found to be an independent predictor of OSA severity. Further research is needed to explore the differences in OSA severity between genders.

### 4.2 Anatomical risk factors

Adenotonsillar hypertrophy contributes to pediatric OSA by narrowing the upper airway (7). However, the link between OSA severity and adenoids and tonsils size remains unclear (27). This study reveals an association between adenoid hypertrophy and moderate-to-severe OSA (OR 2.16; 95% CI, 1.41–3.30). In contrast, there was no significant association with tonsillar hypertrophy (OR 1.00; 95% CI, 0.64–1.56) (Supplementary Table S1). Consistent with prior studies, the severity of obstructive sleep apnea (OSA) correlates with adenoid grade rather than tonsil size (27, 28). Our study found that 75% of children with adenotonsillar hypertrophy exhibited moderate-to-severe OSA, highlighting a stronger association between moderate-to-severe OSA and combined adenoid and tonsil enlargement (OR = 1.58; 95% CI, 1.05–2.36) in comparison to isolated enlargement of either tissue. Similar to previous studies, combined adenotonsillar hypertrophy increases the risk of OSA to a greater extent than isolated adenoids or tonsils hypertrophy and can predict AHI >5 episodes/hour in children (29, 30). This suggests a synergistic effect when both structures are affected.

Obesity contributes to the accumulation of fat around the upper airway and impacts airway patency during sleep (2). Our study found a high prevalence of moderate-to-severe OSA (71%) among obese children, consistent with the previously reported rate of 77% (31). This further highlights the strong association between obesity and severity of pediatric.

Previous studies indicated that craniofacial abnormalities play a significant role in pathogenesis and are associated with the presence and/or severity of OSA (32–34). However, our study did not find a statistically significant association between craniofacial abnormalities and OSA severity, this may be due to the relatively small sample size of affected children. Further research is needed to clarify this potential relationship.

### 4.3 Clinical symptoms of obstructive sleep apnea

Snoring is an important clinical predictor for indicating the necessity to screen for OSA in children (7). Additionally, the frequency of snoring has a positive correlation with an increased risk of severe OSA. In our study, 88.7% of patients with moderate-to-severe OSA experienced frequent snoring. Notably, frequent snoring (>5 nights/week) emerged as the strongest indicator of moderate-to-severe OSA (OR 6.86; 95% CI, 4.40–10.67) (Table 2). Similar to previous studies, children who snore more than half of their sleep time are at a higher risk of having moderate-to-severe OSA (35, 36).

Parents of children with recurrent airway obstruction may observe their children gasping, stopping breathing, awakening during sleep, or frequently changing sleep positions to promote airway patency (2). In this study, awakening during sleep was a significant predictor of moderate-to-severe OSA. Consistent with previous research, stopping breathing during sleep is a strong predictor of pediatric OSA (7, 8, 36). Our study further supports this finding, demonstrating that 66.1% of children with moderate-to-severe OSA exhibited episodes of stopped breathing during sleep.

OSA may cause insufficient sleep, leading to EDS in children (9). However, information regarding the relationship between EDS and severity of OSA remains controversial. Some research indicates a negative correlation, especially among young children (31). While another study found that the frequency of EDS increase along with the severity of OSA. Alexopoulos et al. reported an association between EDS and moderate-to-severe OSA (37). This study had similar findings to our study as we also found a significant association, with 79% of children with EDS having moderate-to-severe OSA, providing additional evidence for EDS as a predictor for moderate-to-severe OSA in children (OR 2.10; 95% CI, 1.28–3.43) (Table 2).

Chronic upper airway inflammation can contribute to pediatric OSA (1). Anti-inflammatory therapies, including intranasal corticosteroids or montelukast, may improve symptoms and AHI in children with mild OSA or those awaiting adenotonsillectomy (38). Despite 91% of children receiving anti-inflammatory therapies in our study, the number of moderate-to-severe OSA cases did not decrease significantly. This suggests that children with mild OSA may benefit more from these therapies than those with moderate or severe OSA. While allergic rhinitis can increase the risk of adenotonsillar hypertrophy and subsequently OSA, recent studies, including our own, have not found a significant association between allergic rhinitis and the severity of OSA (21, 23, 26).

Untreated pediatric OSA may lead to learning and behavioral difficulties, in particular ADHD (20). Previous studies reported a high prevalence (20%–50%) of OSA in children with ADHD, approximately 20% having moderate-to-severe OSA (20, 39). However, our study did not find a significant association between ADHD and moderate-to-severe OSA. This may be due to the limited number of ADHD patients in our study. Further research with larger samples of children with ADHD is necessary to establish a definitive link between ADHD and OSA.

Adenotonsillectomy is recommended for children with moderate to severe OSA (1). These patients are at a higher risk of perioperative complications, including airway obstruction, oxygen desaturation, and unplanned intensive care admission, which necessitate specific perioperative care including close monitoring, specialized anesthetic techniques, and a well-planned postoperative plan (6). Therefore, preoperative diagnosis of at-risk children with moderate-to-severe OSA is essential for facilitating risk stratification and mitigation, ultimately improving patient safety and outcomes (40, 41). However, preoperative PSG is not feasible for all children with suspected OSA due to limited access, long waiting lists, or those requiring emergency surgeries. Furthermore, existing questionnaires for detecting pediatric OSA have a poor correlation with a clear diagnosis of OSA severity (5, 6).

Our study is notable for its innovative use of both RPG and nocturnal oximetry to provide a comprehensive diagnostic assessment of moderate-to-severe OSA in children. Those with multiple risk factors are at an even higher risk, underscoring the importance of prioritizing early sleep studies for timely diagnosis and treatment. In emergency surgical situations, identifying high-risk children is crucial for preoperative planning, anesthetic care, pain management, and perioperative monitoring to ensure safety and reduce complications.

Our study had several limitations. Firstly, there is the potential for nocturnal oximetry to miss milder cases of OSA due to inherent differences in sensitivity and specificity compared to full PSG. However, a study comparing overnight oximetry with simultaneous full PSG in children suspected of OSA reported a positive predictive value of 97% and a negative predictive value of 47%. These findings suggest that oximetry is highly effective in confirming OSA when the results are positive (16).

Secondly, the retrospective design of this study may introduce selection, measurement, and recall biases. To mitigate these risks, we conducted a complete case analysis. Thirdly, as our study was conducted on patients already suspected of having OSA, this may have contributed to the high prevalence of OSA observed and could limit the generalizability of the findings to healthy children without signs or symptoms of OSA. Additionally, we did not evaluate the impact of several previously reported risk factors, including socioeconomic status, breastfeeding, preterm birth, secondhand smoke exposure, and neck circumference (1, 2, 24). Therefore, future research should prospectively examine all potential risk factors in both suspected and non-suspected OSA children to broaden understanding of the potential contributors and applicability to healthy children.

## 5 Conclusions

This study enhances our understanding of pediatric OSA by identifying key risk factors and emphasizing the importance of early identification and assessment. Children aged 1–5 years, those with obesity, adenotonsillar hypertrophy, and specific sleep-related symptoms such as stopped breathing during sleep, frequent snoring, and excessive daytime somnolence are at a higher risk of developing moderate-to-severe pediatric OSA. By recognizing these risk factors, clinicians can tailor treatment and management strategies to improve patient outcomes. Early identification allows for timely interventions, such as surgical interventions or lifestyle modifications, to address OSA and enhance a child's overall health and well-being.

## Data Availability

The raw data supporting the conclusions of this article will be made available by the authors, without undue reservation.

## References

[1] Kaditis AG Alonso Alvarez ML Boudewyns A Alexopoulos EI Ersu R Joosten K Obstructive sleep disordered breathing in 2- to 18-year-old children: diagnosis and management. Eur Respir J. (2016) 47(1):69–94. 10.1183/13993003.00385-2015 26541535

[2] Chang SJ Chae KY. Obstructive sleep apnea syndrome in children: epidemiology, pathophysiology, diagnosis and sequelae. Korean J Pediatr. (2010) 53(10):863–71. 10.3345/kjp.2010.53.10.863 21189956 PMC3004499

[3] Lumeng JC Chervin RD. Epidemiology of pediatric obstructive sleep apnea. Proc Am Thorac Soc. (2008) 5(2):242–52. 10.1513/pats.200708-135MG 18250218 PMC2645255

[4] Brown KA. Outcome, risk, and error and the child with obstructive sleep apnea. Paediatr Anaesth. (2011) 21(7):771–80. 10.1111/j.1460-9592.2011.03597.x 21539679

[5] Kang M Mo F Witmans M Santiago V Tablizo MA. Trends in diagnosing obstructive sleep apnea in pediatrics. Children (Basel). (2022) 9(3):8. 10.3390/children9030306 PMC8947481 35327678

[6] Chandrakantan A Mehta D Adler AC. Pediatric obstructive sleep apnea revisited: perioperative considerations for the pediatric anesthesiologist. Int J Pediatr Otorhinolaryngol. (2020) 139:110420. 10.1016/j.ijporl.2020.110420 33035805

[7] Certal V Catumbela E Winck JC Azevedo I Teixeira-Pinto A Costa-Pereira A. Clinical assessment of pediatric obstructive sleep apnea: a systematic review and meta-analysis. Laryngoscope. (2012) 122(9):2105–14. 10.1002/lary.23465 22886768

[8] Kang KT Weng WC Lee CH Hsiao TY Lee PL Hsu WC. Clinical risk assessment model for pediatric obstructive sleep apnea. Laryngoscope. (2016) 126(10):2403–9. 10.1002/lary.25912 26973061

[9] Tsukada E Kitamura S Enomoto M Moriwaki A Kamio Y Asada T Prevalence of childhood obstructive sleep apnea syndrome and its role in daytime sleepiness. PLoS One. (2018) 13(10):e0204409. 10.1371/journal.pone.0204409 30281638 PMC6169921

[10] Cielo CM Marcus CL. Obstructive sleep apnoea in children with craniofacial syndromes. Paediatr Respir Rev. (2015) 16(3):189–96. 10.1016/j.prrv.2014.11.003 25555676 PMC4454627

[11] Brodsky L. Modern assessment of tonsils and adenoids. Pediatr Clin North Am. (1989) 36(6):1551–69. 10.1016/S0031-3955(16)36806-7 2685730

[12] Gliklich RE Metson R. Techniques for outcomes research in chronic sinusitis. Laryngoscope. (1995) 105(4):387–90. 10.1288/00005537-199504000-00010 7715384

[13] Shen L Lin Z Lin X Yang Z. Risk factors associated with obstructive sleep apnea-hypopnea syndrome in Chinese children: a single center retrospective case-control study. PLoS One. (2018) 13(9):e0203695. 10.1371/journal.pone.0203695 30212502 PMC6136758

[14] Marcus CL Brooks LJ Draper KA Gozal D Halbower AC Jones J Diagnosis and management of childhood obstructive sleep apnea syndrome. Pediatrics. (2012) 130(3):e714–55. 10.1542/peds.2012-1672 22926176

[15] Borrelli M Corcione A Cimbalo C Annunziata A Basilicata S Fiorentino G Diagnosis of paediatric obstructive sleep-disordered breathing beyond polysomnography. Children (Basel). (2023) 10(8):8–9. 10.3390/children10081331 PMC10452996 37628330

[16] Brouillette RT Morielli A Leimanis A Waters KA Luciano R Ducharme FM. Nocturnal pulse oximetry as an abbreviated testing modality for pediatric obstructive sleep apnea. Pediatrics. (2000) 105(2):405–12. 10.1542/peds.105.2.405 10654964

[17] Nixon GM Kermack AS Davis GM Manoukian JJ Brown KA Brouillette RT. Planning adenotonsillectomy in children with obstructive sleep apnea: the role of overnight oximetry. Pediatrics. (2004) 113(1 Pt 1):e19–25. 10.1542/peds.113.1.e19 14702490

[18] Xu Z Wu Y Tai J Feng G Ge W Zheng L Risk factors of obstructive sleep apnea syndrome in children. J Otolaryngol Head Neck Surg. (2020) 49(1):11. 10.1186/s40463-020-0404-1 32131901 PMC7057627

[19] Xiao L Su S Liang J Jiang Y Shu Y Ding L. Analysis of the risk factors associated with obstructive sleep apnea syndrome in Chinese children. Front Pediatr. (2022) 10:3–7. 10.3389/fped.2022.900216 PMC9273047 35832580

[20] Urbano GL Tablizo BJ Moufarrej Y Tablizo MA Chen ML Witmans M. The link between pediatric obstructive sleep apnea (OSA) and attention deficit hyperactivity disorder (ADHD). Children (Basel). (2021) 8(9):2–3. 10.3390/children8090824 PMC8470037 34572256

[21] Lin SY Melvin TA Boss EF Ishman SL. The association between allergic rhinitis and sleep-disordered breathing in children: a systematic review. Int Forum Allergy Rhinol. (2013) 3(6):504–9. 10.1002/alr.21123 23307785

[22] Selvadurai S Voutsas G Propst EJ Wolter NE Narang I. Obstructive sleep apnea in children aged 3 years and younger: rate and risk factors. Paediatr Child Health. (2020) 25(7):432–8. 10.1093/pch/pxz097 33173554 PMC7606157

[23] Tamanyan K Walter LM Davey MJ Nixon GM Horne RS Biggs SN. Risk factors for obstructive sleep apnoea in Australian children. J Paediatr Child Health. (2016) 52(5):512–7. 10.1111/jpc.13120 27329904

[24] Murto KT Zalan J Vaccani JP. Paediatric adenotonsillectomy, part 1: surgical perspectives relevant to the anaesthetist. BJA Educ. (2020) 20(6):184–92. 10.1016/j.bjae.2020.02.007 33456949 PMC7808071

[25] Brockmann PE Koren D Kheirandish-Gozal L Gozal D. Gender dimorphism in pediatric OSA: is it for real? Respir Physiol Neurobiol. (2017) 245:83–8. 10.1016/j.resp.2016.11.010 27890604

[26] Li AM So HK Au CT Ho C Lau J Ng SK Epidemiology of obstructive sleep apnoea syndrome in Chinese children: a two-phase community study. Thorax. (2010) 65(11):991–7. 10.1136/thx.2010.134858 20965935

[27] Nolan J Brietzke SE. Systematic review of pediatric tonsil size and polysomnogram-measured obstructive sleep apnea severity. Otolaryngol Head Neck Surg. (2011) 144(6):844–50. 10.1177/0194599811400683 21493309

[28] Tagaya M Nakata S Yasuma F Miyazaki S Sasaki F Morinaga M Relationship between adenoid size and severity of obstructive sleep apnea in preschool children. Int J Pediatr Otorhinolaryngol. (2012) 76(12):1827–30. 10.1016/j.ijporl.2012.09.010 23021529

[29] Xu Z Cheuk DK Lee SL. Clinical evaluation in predicting childhood obstructive sleep apnea. Chest. (2006) 130(6):1765–71. 10.1016/S0012-3692(15)50899-4 17166994

[30] Kang KT Chou CH Weng WC Lee PL Hsu WC. Associations between adenotonsillar hypertrophy, age, and obesity in children with obstructive sleep apnea. PLoS One. (2013) 8(10):e78666. 10.1371/journal.pone.0078666 24205291 PMC3808373

[31] Bseikri M Zhang J Kirley J Lee C Castillo A Feliciano EMC. Predicting obstructive sleep apnea severity in children referred for polysomnography: use of the pediatric sleep questionnaire and subscales. Sleep Breath. (2023) 27(2):545–52. 10.1007/s11325-022-02647-6 35633476

[32] Xu Q Wang X Li N Wang Y Xu X Guo J. Craniofacial and upper airway morphological characteristics associated with the presence and severity of obstructive sleep apnea in Chinese children. Front Pediatr. (2023) 11:3–8. 10.3389/fped.2023.1124610 PMC10102523 37063671

[33] Lam DJ Jensen CC Mueller BA Starr JR Cunningham ML Weaver EM. Pediatric sleep apnea and craniofacial anomalies: a population-based case-control study. Laryngoscope. (2010) 120(10):2098–105. 10.1002/lary.21093 20824784 PMC4826142

[34] Borrelli M Corcione A Rongo R Cantone E Scala I Bruzzese D Obstructive sleep apnoea in children with down syndrome: a multidisciplinary approach. J Pers Med. (2023) 13(1):5–12. 10.3390/jpm13010071 PMC9862921 36675732

[35] Lewis KC Schroeder JW Jr. Ayub B Bhushan B. Clinical symptoms that predict the presence of obstructive sleep apnea. Int J Pediatr Otorhinolaryngol. (2017) 95:139–44. 10.1016/j.ijporl.2017.02.018 28576523

[36] Raman VT Splaingard M Tumin D Rice J Jatana KR Tobias JD. Utility of screening questionnaire, obesity, neck circumference, and sleep polysomnography to predict sleep-disordered breathing in children and adolescents. Paediatr Anaesth. (2016) 26(6):655–64. 10.1111/pan.12911 27111886

[37] Alexopoulos EI Theologi V Malakasioti G Maragozidis P Tsilioni I Chrousos G Obstructive sleep apnea, excessive daytime sleepiness, and morning plasma TNF-α levels in Greek children. Sleep. (2013) 36(11):1633–8. 10.5665/sleep.3114 24179295 PMC3792379

[38] Kuhle S Hoffmann DU Mitra S Urschitz MS. Anti-inflammatory medications for obstructive sleep apnoea in children. Cochrane Database Syst Rev. (2020) 1(1):Cd007074. 10.1002/14651858.CD007074.pub3 31978261 PMC6984442

[39] Huang YS Chen NH Li HY Wu YY Chao CC Guilleminault C. Sleep disorders in Taiwanese children with attention deficit/hyperactivity disorder. J Sleep Res. (2004) 13(3):269–77. 10.1111/j.1365-2869.2004.00408.x 15339263

[40] Loadsman JA Hillman DR. Anaesthesia and sleep apnoea. Br J Anaesth. (2001) 86(2):254–66. 10.1093/bja/86.2.254 11573670

[41] Patino M Sadhasivam S Mahmoud M. Obstructive sleep apnoea in children: perioperative considerations. Br J Anaesth. (2013) 111(Suppl 1):i83–95. 10.1093/bja/aet371 24335402

