# Supplementary material for: Clinical predictors of moderate-to-severe pediatric obstructive sleep apnea
Source: Front Pediatr. 2024 Oct 25;12:1421467. doi: 10.3389/fped.2024.1421467 (PMC11549671; doi:10.3389/fped.2024.1421467)
Supplement: Supplementary file 1 [file Table1.pdf]

Supplementary Table 1. Univariable and multivariable logistic regression analysis for an independent predictor of moderate-to-severe OSA.

**Characteristics**	**Univariable analysis**				**Multivariable analysis**			
(*n* = 542)	uOR	95% CI		*p*-value	aOR	95% CI		*p*-value
Age 1 – 5 years	5.15	3.73	7.11	<0.001	6.02	3.89	9.33	<0.001
Allergic rhinitis	0.73	0.45	1.16	0.191	0.84	0.45	1.55	0.578
ADHD[1]	2.01	0.86	4.68	0.104	2.23	0.78	6.43	0.136
Obesity	1.42	1.05	1.93	0.023	2.08	1.35	3.20	0.001
Craniofacial abnormalities	0.98	0.50	1.90	0.955	1.44	0.56	3.69	0.446
Adenoid hypertrophy	3.02	2.20	4.13	<0.001	2.16	1.42	3.31	<0.001
Tonsillar hypertrophy	1.92	1.40	2.62	<0.001	1.00	0.64	1.56	0.991
Frequent snoring	15.02	10.4	21.6	<0.001	7.37	4.70	11.57	<0.001
Stopped breathing during sleep	7.15	5.10	10.0	<0.001	2.26	1.45	3.54	<0.001
Awakening during sleep	5.01	3.58	7.03	<0.001	2.00	1.30	3.07	0.002
Excessive daytime somnolence	2.30	1.60	3.30	<0.001	2.03	1.24	3.35	0.005

[1] ADHD, Attention Deficit/Hyperactivity Disorder.
